# Modern wITCHcraft: Conjuring pruritus‐associated gene signatures through microarray profiling

**DOI:** 10.1111/jdv.70287

**Published:** 2026-02-25

**Authors:** Simon M. Mueller

**Affiliations:** ^1^ Department of Dermatology, University Hospital Basel University of Basel Basel Switzerland

Pruritus (synonymous with itch) is the most common symptom among dermatology patients, and when chronic (≥6 weeks), it may evolve into a burdensome, multidimensional condition that is often challenging to treat, as summarized in the recent European guideline.^1^

The single‐authored article by Alsabbagh entitled *The expression of pruritus‐associated genes in seven skin diseases: Evidence from microarray* ^2^ adds a new piece to the complex puzzle of pruritus pathophysiology. The author's analysis of microarray datasets from the Gene Expression Omnibus database, combined with pruritus‐associated gene sets curated from the Gene Set Enrichment Molecular Signatures database, provides an innovative view through a genomic lens. While microarrays themselves are well established, the novelty lies in the methodological approach of leveraging publicly available databases and integrating multiple pruritic conditions in the same analysis. This strategy enables the detection of core pathways potentially underlying pruritus across conditions, while simultaneously revealing unique gene expression patterns that may guide disease‐specific therapeutic interventions, as illustrated in this publication also for acne, lupus, alopecia areata and rosacea‐diseases that are not typically considered highly pruritic.

For example, *CARD14* and *PKP1* were upregulated only in atopic dermatitis and psoriasis, while *IL7R* was upregulated across all seven conditions including the four mentioned above. However, gene upregulation does not necessarily imply pathological relevance. IL7 deficiency has previously been shown to exacerbate atopic dermatitis in a murine model,^3^ suggesting that *IL7R* upregulation may represent a compensatory response. Likewise, Alsabbagh reported normal IL‐7 levels in lesional skin of acne and lupus, indicating that *IL7R* may not constitute a meaningful therapeutic target for itch in these diseases.

The three witches in Shakespeare's *Macbeth* declare ‘The charm's wound up’, (Act 1, Scene 3), signalling that the spell is cast and their enticing prophecies for the future are set in motion‐a fitting metaphor for the seductive, yet potentially misleading power of such ‘wITCHcraft’ datasets. Microarrays provide only static snapshots of molecular states and constitute just one element within a much broader *omics* ecosystem. This landscape encompasses targeted transcriptomic platforms such as NanoString, bulk and single‐cell RNA sequencing, spatial transcriptomics and integrative multi‐omics strategies that combine transcriptomics with genomics, epigenomics, proteomics, metabolomics and immune profiling. Collectively, these approaches offer complementary information across multiple biological scales, ranging from tissue‐level averages to cell type‐specific and spatially resolved molecular programs, thereby enabling a more nuanced understanding of pruritus mechanisms. While these technologies are powerful for hypothesis generation and for constructing conceptual frameworks that inform precision medicine strategies, they also share important limitations: these include susceptibility to technical and batch‐related artefacts, a predominance of associative rather than causal inference, limited temporal resolution, and‐most critically‐a frequent lack of direct functional validation linking molecular signatures to clinically relevant biology. Accordingly, upregulated genes should be selectively targeted to validate their pathological relevance, while downregulated genes may be experimentally stimulated to evaluate their potential antipruritic effects.

Finally, epigenetic regulation, dynamic changes in gene expression over the disease course, as well as the intricate interplay between itch perception, psychological factors, environmental influences, coping strategies and access to therapeutic options remain equally critical components of the larger pruritus puzzle.

## CONFLICT OF INTEREST STATEMENT

SMM is a member of the EADV Task Force Group Pruritus and is/was a consultant and has received speaking fees and/or grants from and/or served as an investigator in clinical trials for Sanofi‐Aventis AG, Galderma SA, Janssen‐Cilag AG, LEO Pharmaceutical Products Sarath, Amgen, Incyte, Novartis Pharma AG and UCB Pharma.

## Data Availability

Data sharing is not applicable to this article as no new data were created or analysed in this study.
